# Empathy’s Role in Engineering Ethics: Empathizing with One’s Self to Others Across the Globe

**DOI:** 10.1007/s11948-024-00512-1

**Published:** 2024-11-25

**Authors:** Justin L Hess

**Affiliations:** Neil Armstrong Hall of Engineering, Room 1331, 701 W. Stadium Avenue, West Lafayette, IN 47907 USA

**Keywords:** Empathy, Microethics, Macroethics, Engineering ethics education

## Abstract

Engineers make decisions with global impacts and empathy can motivate ethical reasoning and behavior that is sensitive to the needs and perspectives of stakeholders across the globe. Microethics and macroethics offer two frames of reference for engineering ethics education, but different dimensions of empathy play distinct roles in micro- and macroethics. Microethics emphasizes individual responsibility and interpersonal relationships whereas macroethics emphasizes societal obligations and impacts. While empathy can support ethical reasoning and behavior for each, in this paper I argue that affective empathy plays a primary (but not exclusive) role in microethics whereas cognitive empathy plays a primary role in macroethics. Gilligan’s and Kohlberg’s theories of moral development are used to further depict how affective empathy aligns with care (depicted as an interpersonal phenomenon) and how cognitive empathy aligns with justice (depicted as a systems-focused phenomenon), thus positioning these ethical principles as playing primary (but again, not exclusive) roles in micro- and macro-ethics, respectively. Building on these ideas, this study generates a framework that describes and visualizes how empathy manifests across six frames of reference, each of which are increasingly macro-ethical in nature: self, team, operators, participants, bystanders, and future generations. The paper describes how proxy stakeholders can be identified, developed, and leveraged to empathize with stakeholder groups. Taken together, the manuscript seeks to clarify the role of empathy in engineering ethics and can enable engineering students to better empathize with the range of stakeholders impacted by engineering decisions, ranging from one’s self to stakeholders across the globe. The intrapersonal understandings and motivations that students generate by empathizing across six frames of reference can facilitate ethical reasoning processes and behaviors that are more inclusive and comprehensive.

## Introduction

In engineering education, microethics and macroethics are oft-used theoretical frameworks for fostering ethical reasoning among students (Martin et al., 2021; Rottmann & Reeve, 2020). Microethics and macroethics draw attention to different facets of professionalism, where microethics prioritizes individual and interpersonal aspects of professionalism and macroethics centers collective social responsibilities of the engineering profession and societal impacts of engineering work (Herkert, 2001, 2005). Given the role of empathy in ethical decision-making writ broadly (Gibbs, 2013) and in engineering specifically (Hess et al., 2019; Walther et al., 2020), this paper aims to depict how empathy manifests in microethics and macroethics. As this paper theorizes, empathy is valuable to both microethics and macroethics but the primary way that empathy manifests and informs ethical reasoning or action is distinct in each.

For ABET (2024) accreditation, engineering programs must develop within students “an ability to recognize ethical and professional responsibilities in engineering situations and make informed judgments, which must consider the impact of engineering solutions in global, economic, environmental, and societal contexts.” Thus, engineers must engage in ethical decision-making processes that span individual considerations (e.g., how ought I act?) to global impacts (e.g., how do my decisions impact stakeholders across the world?). Empathy draws attention to others’ needs, values, and perspectives and can invoke a salience effect, thus leading one to include other perspectives into one’s decision-making processes (Oxley, 2011). The framework offered in this paper serves as a heuristic for engineers to consider stakeholders across the globe, and the associated discussion will identify how empathy encourages the incorporation of such perspectives into one’s decision-making processes. Thus, the framework will offer instructors with a tool to guide students’ ethical deliberation and, in turn, realize ABET’s (2024) ethics-focused student outcome.

The aims of this paper are (1) to depict how different ways of conceptualizing empathy align with and can guide micro-ethical and macro-ethical reasoning or behavior and (2) to describe a practical framework that engineering instructors can leverage to encourage student empathy with stakeholders across the globe. To achieve these aims, the paper articulates how microethics and macroethics correspond with moral developmental theories which emphasize the principles of care (Gilligan, 1993) and justice (Kohlberg, 1984), respectively. While the paper focuses on engineering, the framework is transferrable to other disciplines where ethics requires interpersonal engagement and the incorporation of societal perspectives.

Given the culture of disengagement in engineering programs (Cech, 2014), the tendency of engineers to act in ways that prioritize engineers over society (Lambrinidou et al., 2014), and the interpretive nature of engineering codes of ethics (Smith, 2021), there is a need for resources that can motivate engineers to seriously consider stakeholders whom they may neglect, including but not limited to innocent bystanders (Hoffman, 2000). The argument presented in this work is that empathy can serve to motivate engineers to account for stakeholders’ needs, including oneself and others, and including stakeholders near and far whom engineers otherwise may neglect to consider or prioritize. To support this argument, the paper addresses five questions: 

**Q1**: How do microethics and macroethics manifest in engineering?

**Q2**: What is empathy and why is it important to ethics in engineering, in general?

**Q3**: How does empathy align with micro-ethical and macro-ethical frames of references?

**Q4**: How can six frames of references inform who engineers strive to empathize with? 

**Q5**: How can engineers empathize with stakeholders ranging from one's self to others across the globe?

Exploring extantliterature regarding scholarly stances on Q1 and Q2 sets the stage for Q3, where this paper considers relational patterns between micro/macro ethics, affective/cognitive empathy, and care/justice. Thus, responses to Q3 represent the key theoretical contributions of this paper. In turn, the section devoted to Q4 offers a practical framework that instructors may leverage in their curriculum towards realizing the social and global aspirations identified in ABET’s (2024) ethics-related outcome. Finally, the section devoted to Q5 draws attention to how engineers can develop and leverage proxy stakeholders to empathize with stakeholder groups. Here, design thinking literature is leveraged to define how personas can inform the generation of such proxies.

Taken together, this manuscript seeks to bolster efforts towards promoting empathy, microethics, and macroethics in engineering ethics education. To this end, this paper employs multiple dualisms, including micro/macro ethics (Herkert, 2005), affective/cognitive empathy (Clark et al., 2019), and care/justice (Hoffman, 2000). This is not the first work to elaborate on these dualisms. The novel addition of this work is clarifying the role of empathy within these previously described framing devices as well as shared patterns or commonalities across the dualisms. Importantly, as in each of these prior works, the dualisms themselves interplay, inform each other, and can play supporting roles. More importantly, the dualisms serve to facilitate an understanding of how distinct dimensions of empathy manifest in engineering ethics.

## Q1: How do Microethics and Macroethics Manifest in Engineering?

Herkert (2005) extended and popularized the framing offered by Ladd (1980) of microethics and macroethics. As Herkert described, “John Ladd subdivides engineering ethics into ‘microethics’ or ‘macroethics’ depending on whether the focus is on relationships between individual engineers and their clients, colleagues and employers, or on collective social responsibility of the profession” (p. 374). Herkert expands this definition by writing:

Engineering ethics can be viewed from three frames of reference—individual, professional and social—which can be divided into “microethics” concerned with ethical decision making by individual engineers and the engineering profession’s internal relationships, and “macroethics” referring to the profession’s collective social responsibility and to societal decisions about technology. (Herkert, 2005, p. 374)

Microethics involves understanding and upholding canons in one’s practice. Micro-ethical concerns may be “professional” in nature, but they may also involve ethical decision-making irrespective of one’s professional code. Thus, microethics includes “internal relationships” (p. 374) and myriad associated interpersonal considerations, such as listening or responding to peers or colleagues to make more holistic ethical judgments or negotiate disagreements regarding how a team ought to respond to an ethical dilemma. Indeed, listening has been described as critical to engineering ethics (Lambrinidou et al., 2014), albeit, it has historically been missing from engineering education (Leydens & Lucena, 2009).

Macroethics draws attention to societal impacts and responsibilities of a profession. In engineering, macroethics may involve attending to the needs of a specific well-defined user group or a broad ill-defined stakeholder group. LaPatin et al. (2021) identified eight types of macro-ethical issues which students perceived as applicable to civil engineers. These included ascertaining how to respond to environmental issues, promoting economic growth, and improving and building infrastructure. Arguably, the Sustainable Development Goals (United Nations General Assembly, 2015) include macro-ethical global issues (e.g., Quality Education for all, Clean Water and Sanitation) which, like all aspects of sustainability, are salient to engineers (Harrison & Kelley, 2019). Listening (a professional skill) to community voices can inform the development of sustainable solutions for communities (Leydens & Lucena, 2009; Bielefeldt, 2020); thus, there is a relationship between micro-and-macro ethics.

Microethics has historically dominated engineering ethics instruction (Herkert, 2005), but recent years have potentially seen a more concerted focus on macroethics. For example, Swan et al. (2019) examined engineering ethics cases studies and identified a shift in the foci of cases from “specific micro-ethical dilemmas” to “more generalized, macro-ethical ‘best practices’ over the past few decades.” Rottmann and Reeve (2020) identified a similar pattern in engineering ethics education literature. They noted, however, that microethics and macroethics each bring “advantages and disadvantages.” For example, micro-ethical cases tend to “be more accessible and relatable to students with limited work experiences” and macro-ethical cases “help students consider the consequences of engineers’ work” (p. 149).

Importantly, ethics instruction need not (and should not) choose between microethics or macroethics. Herkert (2005) called for instruction that “integrate[s] micro-ethical and macroethical concerns” (p. 382). Such integrative strategies were offered by McAninch (2023) for engineering ethics education. For example, McAninch shared that “educators can encourage students to approach these [ethical] problems from the perspective of an individual professional working within the kind of institutional, hierarchical environment they are likely to enter” (p. 20). McAninch thus encouraged educators to prompt engagement with individual and organizational perspectives.

The distinction between microethics and macroethics identifies where one directs their attention. This is not to suggest that micro-ethical practices cannot have macro-ethical impacts, nor vice versa. For example, consider an engineering intern who needs to discard a large volume of toxic coolant. Their supervisor tells them “to dump half of the used coolant down the drain” and assures the student that “toxins settle at the bottom” and will lead to no human or environmental health damages (Pritchard, 1992). Regardless of the illegality of toxic dumping, should the intern abide by their supervisor’s direction, there is a significant risk of downstream impacts to the environment and on human health. While this case is fictitious (which is not to suggest that it could not occur), a real-world historic case of the Ford Pinto highlights how micro-ethical decisions can lead to large-scale harms. In this case, Ford chose to prioritize the reduction of production costs over driver safety; Ford engineers failed to pushback or whistle-blow despite safety concerns (De George, 2017). The decisions that Ford made involved relatively predictable outcomes and a lack of transparency. The ethicality of Ford engineers’ actions (or lack thereof) are debatable (De George, 2017); they may be deemed ethical insofar as the Ford engineers did what was expected of them in the organization but potentially unethical insofar as the same engineers failed to prioritize avoiding public harm (i.e., macro-ethical impacts). Importantly, today we reflect on the Ford case with hindsight and can clearly discern that there were predictable macro-ethical outcomes. Yet, given the complexity of engineering systems, many micro-ethical decisions can have uncertain or unpredictable downstream macro-ethical impacts (Perrow, 1999), especially during the time of the event.

McAninch (2023) elaborates on the relationship between microethics and macroethics by calling attention to how professional norms can dictate micro-ethical action. As McAninch argued, “Microethics and macroethics may be conceptually independent, in the sense that one’s actions could be in full compliance with micro-ethical standards and yet still generate macro-ethical concerns” (p. 20). Yet, if we consider macro-ethical impacts to be an important aspect of engineers’ ethical responsibility, including but not limited to when outcomes are predictable, then it follows that micro-ethical decisions must strive to account for macro-ethical impacts. This idea is codified in the first canon from NSPE: “Engineers, in the fulfillment of their professional duties, shall hold paramount the safety, health, and welfare of the public.” In short, NSPE codified the import of accounting for macro-ethical impacts of engineered works and this clause is included in most modern codes of ethics for engineering.

Micro-ethical decisions have macro-ethical impacts; similarly, macro-ethical decisions can have micro-level impacts. Should engineers prioritize macro-ethical thinking alone, then their ethical practice would be incomplete. In the case of Ford, the engineers’ cost-benefit analysis seemed to neglect downstream micro-level considerations, such as “the human suffering of the victim[s] and his or her [their] family” (De George, 2017, p. 8); one might argue that such analysis lacked empathy for individuals. The Ford Pinto case is like other failures that arise when engineers deal with complex technological systems. As Perrow (1999) argued, “system-related production pressures” can often “defeat safety improvements” (p. 63). Post-hoc, such system-wide (i.e., macro-ethical) failures can lead to additions or shifts in policies, rules, or codes themselves, thus informing micro-ethical norms of practice.

Professionalism was central to Herkert’s (2005) framing of microethics and macroethics. Professional ethics are made explicit in engineering codes of ethics (Davis, 1991) and can inform whether microethics and/or macroethics is emphasized in engineering ethics education. More importantly, such codes can inform engineers’ ethical practice. For example, the Fundamentals Exam emphasizes “micro-ethical questions,” and such micro-ethical considerations were historically centered in US engineering codes of ethics (McAninch, 2023). Swan et al. (2019) found that as the NSPE code of ethics incorporated more macro-ethical considerations, there was a simultaneous increase of macro-ethical considerations in engineering ethics case studies. This aligns with Hudspith (1991), who noted a lack of macroethics in engineering codes of ethics and, as a result, a lack of macroethics in engineering instruction.

While Hudspith (1991) argued that current codes emphasized “the conduct of the individuals in specific situations that arise in the day-to-day practice of engineering” (p. 209), Hudspith found that engineering codes of ethics at the time incorporated macro-ethical suggestions. Hudspith cited examples offered by the “Model Ethics Code” presented by Unger (1982), such as: (1) “[Engineers should] inform themselves and others, as appropriate, of the consequences, direct *and indirect*, immediate and *remote*, of projects they are involved in,” and (2) “[Engineers should] keep their professional skills up to date and be *aware of current events* and *societal issues* pertinent to their work” (p. 210). Macro-ethical considerations are now central to many engineering codes of ethics, albeit, there remains room to improve such codes in certain areas, such as social justice (Riley & Lambrinidou, 2015) and equity (Rottmann & Reeve, 2020).

Micro-ethical and macro-ethical frameworks guide one to ask different types of questions (Herkert, 2005). In the context of professional responsibility, a micro-ethicist might ask, “What ought I do as an individual or as a professional engineer?” A macro-ethicist, on the other hand, may ask, “What ought we do as an engineering profession or as an engineering organization to resolve this ethical issue?” In the context of how engineering decisions impact others, micro-ethicists might ask, “How does what I say and do affect another?” whereas macro-ethicists may instead ask, “How does what I say and do impact groups, communities, regions, nations, or the earth?” Empathy can motivate engineers to address these questions in ways that center select stakeholders, but how empathy manifests may vary based on whether engineers consider micro-ethical or macro-ethical questions. To better understand how empathy manifests in distinct ways to support ethical reasoning or action, we must unpack what empathy is.

## Q2: What is Empathy and Why is it Important to Ethics in Engineering, in General?

Empathy is a multi-dimensional phenomenon comprised of at least eight distinct “concepts” (Batson, 2009). Clark et al. (2019) argued that there are three overarching empathy dimensions: cognitive empathy, affective empathy, and behavioral empathy. These dimensions are comprised of multiple empathy concepts. For example, projection (an empathy concept) is a type of cognitive empathy, while empathic concern (another empathy concept) is a type of affective empathy. Batson’s eight empathy concepts are presented in the order Batson presented them in Table 1, and the shorthand for the terms was previously presented in Hess and Fila (2016). The final column identifies how the empathy concepts align with affective and cognitive empathy.

**Table 1:** Categorization of eight empathy concepts as affective or cognitive empathy (developed based on Batson, 2009 and Hess & Fila, 2016)

Empathy Type (from Hess & Fila, 2016)	Quotes (taken from Batson, 2009)	Empathy Dimension
1. Empathic accuracy	“Knowing another person’s internal state, including his or her thoughts or feelings.”	Cognitive Empathy
2. Motor mimicry	“Adopting the posture or matching the neural response of an observed other.”	Affective Empathy
3. Emotional contagion	“Coming to feel as another person feels.”	Affective Empathy
4. Projection	“Intuiting or projecting oneself into another’s situation.”	Cognitive Empathy
5. Perspective-taking: imagine-other	“Imaging how another is thinking or feeling.”	Cognitive Empathy
6. Perspective-taking: imagine-self	“Imagining how one would think and feel in the other’s place.”	Cognitive Empathy
7. Empathic distress	“Feeling distress at witnessing another person’s suffering.”	Affective Empathy
8. Empathic concern	“Feeling for another person who is suffering.”	Affective Empathy

Cognitive empathy connotates the process or outcome of thinking of or as another, affective empathy involves feeling for or internalizing another’s emotional state, and behavioral empathy involves interpersonal events that results from empathy’s cognitive and affective dimensions (Clark et al., 2019). Thus, cognitive empathy and affective empathy can manifest individually or co-manifest to support empathic behaviors. While cognitive and affective empathy can interact and support each other, they are comprised of unique empathy concepts (Batson, 2009) which manifest distinctly (Davis, 2006). For example, one can consider the perspective of an individual (i.e., engage in perspective-taking: imagine-other) but lack empathic distress, and thus feel no impulse to act upon this understanding (Hoffman, 2000). Conversely, one can feel concerned for the needs of another but fail to consider their perspectives or accurately understand their needs.

In social neuroscience literature, affective responses and rational cognitions offer two distinct ways of operationalizing empathy (Batson, 2009). Whether empathy is a behavior or *leads* to behavior is a source of tension among theorists (Cuff et al., 2016). In Table 1, and throughout this paper, empathy will be defined as a cognitive process (i.e., cognitive empathy) or affective experience (i.e., affective empathy). In this framing, all eight of Batson’s empathy concepts are represented – for example, empathic distress, empathic concern, emotion congruence, and motor mimicry are each “concepts” associated with affective empathy. Conversely, empathic accuracy, projection, perspective-taking: imagine-other, and perspective-taking: imagine-self are each associated with cognitive empathy.

Cognitive and affective empathy can operate together or in isolation to motivate empathic behavior (Clark et al., 2019). In this spirit, Davis (2006) depicted how empathic processes (including affective experiences and cognitive processes) can contribute to intrapersonal outcomes (such as accurately understanding others’ views or behaviors) and interpersonal responses (such as engaging in helping behavior or aggression). In Davis’s (2006) model, empathy can even lead to the development of intrinsic motivations which, in turn, can motivate future empathic processes, experiences, and behaviors. In this sense, empathy itself can inspire empathy.

Individual authors may prioritize one empathy dimension or concept but recognize that the dimensions/concepts inter-relate and inform each other (Batson, 2009; Clark et al., 2019; Hess & Fila, 2016). For example, Hoffman described cognitive and affective empathy, but deemed empathic distress as the primary determinant of prosocial behavior. Like Hoffman, Oxley (2011) regarded emotional congruence to be a primary antecedent of *being empathic*, and both argued that emotional congruence leads to more accurate understandings of another’s perspective. Similarly, de Waal (2009) suggested that affective empathy (which he considered an internal, subconscious, and automatic experience) undergirded and informed cognitive empathy. Each of these theorists thus depicted affective empathy as motivating or informing cognitive or behavioral empathy.

In the context of engineering, empathy can help engineers develop stronger relationships (Walther et al., 2020), communicate effectively (Walther et al., 2012), engage in ethical reasoning (Hess et al., 2017, 2019), and prioritize social justice (Cartabuke et al., 2019; Naphan-Kingery et al., 2019). However, like Herkert’s (2005) individual, social, and professional frames, empathy can manifest in distinct ways depending on one’s frame of reference. For example, at the micro-ethical level, empathy plays a primary role in supporting interpersonal relationships. Conversely, at the macro-ethical level, empathy can prompt one to take a “societal perspective” (Strobel et al., 2013) and, in turn, pursue behaviors that benefit society holistically (Walther et al., 2017).

It is likely that engineers will not interact with all stakeholders impacted by their decisions. When lacking direct stakeholder interaction, cognitive empathy can facilitate the integration of stakeholder considerations beyond those with whom the engineer may interact. Social perspective-taking is essential for post-conventional ethical reasoning in Kohlbergian models of moral development (Kohlberg, 1976). Similarly, Gibbs (2013) argued that ethical decisions must be vetted through social perspective-taking, wherein decisions can be deemed justifiable insofar as they are reversible, or appropriate for both oneself and others to engage in. Thus, perspective-taking can reciprocally inform affective empathy which, in turn, can provide the motivational impulse afforded by empathic distress (Hoffman, 2000).

A prominent framework for defining empathy in engineering comes from Walther et al. (2017). These authors provide a non-hierarchical “model of empathy for engineering” which depicts empathy as a “learnable skill” (p. 134), “practice orientation” (p. 135), and “professional way of being” (p. 137). The skill dimension connotates that select empathy skills can manifest if prompted, and through continuous prompting, one can learn to become more empathic (at least as it pertains to the practiced skills). The orientations “serve to contextualize and orient the empathic skills in professional engineering settings” (p. 135). Finally, the “being” dimension discusses the alignment between “value commitments” that permeate engineering practice, including but not limited to in the domain of ethics, and how such commitments themselves can inform the manifestation of empathic ways of being (p. 137).

Components of Walther and colleagues’ (2017) model align with Batson’s (2009) empathy concepts (refer to Table 1). For example, empathy as a skill includes five components. The first is “affective sharing,” which is like (but not synonymous with) Baton’s emotional contagion. The second is “perspective taking,” which is aligned with two distinct but related perspective-taking concepts in Batson’s model. The third is “self and other awareness,” which is represented by Batson’s differentiation between imagine-self and imagine-other perspective-taking. Fourth, “emotion regulation” is aligned with Batson’s empathic distress in that emotion regulation enables one to ensure that one can continue attending to others’ perspectives when one experiences significant emotional distress (Hoffman, 2000). Finally, “mode switching” calls attention to the shift between what Walther et al. (2017) describe as “empathic and analytic cognitive mechanisms” (p. 135). In part, mode switching connotates switching between affective and cognitive empathy components. Thus, there is strong overlap between Batson’s (2009) empathy types and Walther et al.’s (2017) learnable skills. Taken together, the two works highlight that empathy comprises (or at least depends upon) even more than eight concepts.

In addition to depicting empathy as a skill, the Walther et al. (2017) model draws attention to critical facets undergirding or informing the manifestation of empathy concepts (i.e., orientations) as well as potential outcomes or behaviors enacted by empathic individuals (i.e., ways of being). In part, the orientations (i.e., “epistemological openness,” “micro to macro focus,” “reflective value awareness,” and “commitment to value pluralism”) align with *antecedents* to empathic processes described in Davis’s (2006) organizational model of empathy, which include two groups: individual and situational factors. Thus, both aspects of an individual (e.g., their epistemological openness) and the situation (e.g., classroom expectations) inform empathy’s manifestation. Similarly, Walther and colleagues’ (2017) “ways of being” empathic in engineering (i.e., “dignity and worth of all stakeholders,” “service to society,” and “engineers as whole professional”) connotate antecedents to empathy’s manifestation *as well as* outcomes resultant from empathic processes. Indeed, as the authors argue, empathy as a way of being a whole professional hearkens to the need of engineers “to engage with large ethical commitments and moral principles” (p. 140).

The ways of operationalizing empathy presented here each suggest that empathy can inform ethical reasoning and ethical behavior. As the next section depicts, the most relevant empathy dimensions can vary based on whether an engineer’s focus is individualistic or interpersonal in nature (i.e., micro in nature) versus systemic (i.e., macro in nature). Specifically, affective empathy concepts (referenced by Oxley and Hoffman) involve or draw attention to the emotions between a self and another, whereas cognitive empathy (including societal perspective-taking referenced by Gibbs and Kohlberg) finds one imagining the views, needs, and feelings of societal groups or system-level norms. The following section follows this line of reasoning and situates these considerations in two moral developmental frameworks.

## Q3: How does Empathy Align with Micro-ethical and Macro-ethical Frames of Reference?

Oxley (2011) depicted empathy as necessary for ethical decision-making and behavior. Oxley suggested that empathy invokes a salience effect that prompts moral deliberation, wherein the reasoner aspires to accurately account for others’ perspectives. Like Hoffman, Oxley suggested that empathy’s epistemic functions include cognitive empathic processes but emphasized that for this salience effect to become actualized, the affective components of empathy must manifest. Hoffman similarly emphasized affective empathy as central to his theory of moral development, but Hoffman argued that cognitive empathy can inform affective empathy and thus become primary in empathic arousal, especially when stakeholders are far away. Moreover, Hoffman argued that empathy may manifest in distinct ways when considering the principles of care and justice. This section builds on these considerations and (1) introduces care-based and justice-based moral development frameworks, (2) depicts the role of empathy within each, and (3) describes how empathy dimensions (affective and cognitive) and moral principles (care and justice) align with microethics and macroethics.

Care-based frameworks depict ethically ideal interpersonal ways of being (Gilligan, 1993; Tronto, 1993). In this sense, Gilligan’s care-based moral development frameworks aligns with micro-ethical reasoning and practice. While microethics is commonly introduced in engineering curriculums (Herkert et al., 2015; Rottmann & Reeve, 2020), it often is introduced with a focus on individual responsibility. However, interpersonal interactions beyond one’s professional code are also part of the micro-ethical domain. Drawing further on Gilligan’s care-based moral development framework and connecting this to empathy, more comprehensive stages of moral development exhibit growth beyond a lack of empathy wherein the individual focuses primarily on the ego or self, to an emphasis on caring for another by considering and responding to another’s needs, to an emphasis on the self/other in relation and attending to the needs and perspectives of both self and other (Gilligan, 1993). Like microethics, these considerations emphasize the self and relational considerations, which is not to suggest that societal norms or expectations become absent; rather, they are not the focus.

Empathy as traditionally conceived (i.e., as a one-to-one phenomenon) lends itself to interpersonal and micro-ethical issues. However, when we situate empathy in engineering, it involves accounting for a “societal perspective” (Strobel et al., 2013) or engaging in “holistic service to society” (Walther et al., 2017). Segal’s (2011) model of social empathy is particularly helpful here, as it draws attention to the interplay between individual understandings and group inferences. Segal’s social empathy model includes three components: (1) Empathy towards individuals, including “affective response/mirroring” and “cognitive processing” (p. 267), (2) “Contextual understanding,” or an understanding of how context informs empathy’s manifestation (p. 270), and (3) “Social responsibility,” or how one ought to use their empathic understanding to make “macro-level” ethical decisions (p. 271). For Segal, accounting for “historical backgrounds,” “systemic conditions,” and inequities therein was critical for ensuring that empathy leads to social justice. Social empathy has also been empirically studied in engineering education, with findings suggesting that engineering students who have previously experienced suffering are more likely to exhibit empathy and, in turn, an equity ethic (Naphan-Kingery et al., 2019).

Social empathy involves developing individual and contextual understandings, which then informs justice-oriented actions. Social empathy thus aligns with post-conventional modes of ethical reasoning in Kohlbergian and neo-Kohlbergian theories of moral development (Kohlberg, 1976; Rest et al., 2000), specifically in its emphasis on system-level considerations. Kohlberg’s most comprehensive ethical reasoner defines right in terms of “universal principles of justice.” Herein, “the equality of human rights and respect for the dignity of human beings as individual persons” is a primary concern (p. 176). In Kohlberg’s (1976) theory, an individual’s moral development process exhibits invariant progression through six stages of moral development, each of which are associated with a distinct “social perspective[s]” that an individual prioritizes (note: neo-Kohlbergian’s denounce the invariant stage-sequence, see Rest et al., 2000):

1. “Egocentric point of view,” where the individual emphasizes self-interest. (p. 174)
2. “Concrete individualistic perspective,” where the individual views others as having their own self-interests, and views what is “right” to be “relative” (p. 174).
3. “Perspective of the individual in relationships with other individuals,” where one becomes “aware of shared feelings, agreements and expectations which take primacy over individual interests” and enacts “Golden Rule” logic. (p. 174)
4. “Differentiates societal point of view from interpersonal agreement or motives,” where one strives to take “the point of view of the system that defines roles and rules” (p. 175).
5. “Prior-to-society perspective,” where an individual takes the “perspective of a rational individual aware of values and rights prior to social attachments” (p. 175).
6. “Moral point of view,” where one takes on the perspective of “any rational individual recognizing the nature of morality or the fact that persons are ends in themselves and must be treated as such” (p. 176).

In brief, in Kohlberg’s theory, as one develops more comprehensive ethical reasoning abilities, they move from an egocentric self-centered perspective-taking tendency (i.e., stages 1 and 2), towards considering interpersonal relationships (i.e., stage 3), to a societal perspective-taking tendency (i.e., stage 4). The most comprehensive ethical reasoners will emphasize moral principles (namely, justice) while perspective-taking. Here, empathy still involves attending to the thoughts and feelings of others but the “other” also involves system-level considerations (stage 4), followed by rights of all individuals (stage 5) and justice (stage 6).

While Kohlberg’s framework prioritizes justice-oriented thinking, which is largely aligned with Rawlsian justice (Rawls, 1999, originally 1972), Gilligan’s (1993) framework emphasizes the principle of care. Gilligan’s most comprehensive ethical reasoners emphasize care for oneself and others and draws attention to self-other relationality. As Gilligan (1993) describes:

In this [moral development] sequence, an initial focus on caring for the self in order to ensure survival is followed by a transitional phase in which this judgment is criticized as selfish. The criticism signals a new understanding of the connection between self and others which is articulated by the concepts of responsibility. […] At this point [i.e., in the second perspective], the good is equated with caring for others. However, when only others are legitimized as the recipients of the woman’s care, the exclusion of herself gives rise to problems in relationships, creating a disequilibrium that initiates the second transition. […] The third perspective focuses on the dynamics of relationships and dissipates the tension between selfishness and responsibility through a new understanding of the interconnection between other and self. Care becomes the self-chosen principle of a judgment that remains psychological in its concern with relationships and response but becomes universal in its condemnation of exploitation and hurt. (p. 74)

Gilligan’s framework emphasizes that one ought to recognize their responsibility to both oneself and others. Both Gilligan and Kohlberg’s frameworks share a commonality in that both prioritize a principle: justice in the case of Kohlberg and care in the case of Gilligan. This is not to say that these authors view the competing principle as irrelevant, but rather prioritize one over the other. As Hoffman (2000) stated:

Advocates of one principle, caring or justice, accept the other as important but subordinate, especially Kantians who argue that judgments based on individual welfare are not justice judgments unless the welfare is seen as a matter of a ‘right’ that the individual has. Otherwise, caring issues are personal (not subject to legitimate social control), involve affective rather than rationally based decisions, pertain to particular victims and are thus not universal, and lack the formal properties of justice obligations. Caring is therefore logically subordinate to justice obligations [for Kantians] in which the two conflict. (Hoffman, 2000 p. 223)

Due to their prioritization of each principle (care versus justice), it follows that Gilligan and Kohlberg’s frameworks more closely comport with affective and cognitive empathy, respectively. For Hoffman (2000), caring actions result from empathic distress (an affective empathy concept, refer to Table 1). Such feelings may be automatically activated or cognitively induced; thus, cognitive empathy can lead to affective empathy, but empathic distress (at least for Hoffman) plays the key function in empathic behavior. In this same spirit, care and affective empathy more closely align with microethics by drawing attention to how one ought to act in ways that involve caring for the self and others. On the other hand, as depicted in the above quote, justice (at least as described by Kantians) is largely a rationale enterprise and thus is primarily activated through cognitive empathy. Similarly, Kohlbergian’s view of justice builds on systems-level perspective-taking, thus aligning more closely with macroethics.

Importantly, these moral development frameworks should be viewed as alternative moral development pathways. Gilligan even supported Kohlberg’s stage theory (Jorgensen, 2006). Likewise, care is important (even if not the priority) in Kohlberg’s framework (Hoffman, 2000). Thus, while affective empathy, care, and microethics align, as do cognitive empathy, justice, and macroethics, each of these aspects can inform all other aspects. For example, as highlighted in Q1, micro-ethical decisions can have macro-ethical impacts and vice versa. Moreover, as highlighted in Q2, cognitive empathy can inform affective empathy and vice versa (Hess & Fila, 2016), such as through purposeful mode switching (Walther et al., 2017). The next two sections build on these considerations and strive to provide a framework for employing empathy in engineering, specifically as stakeholders move from near to far away, and associated ethical issues largely move from micro-ethical to macro-ethical in nature.

## Q4: How can Six Frames of Reference Inform Who Engineers Strive to Empathize with?

As engineers must consider stakeholders and stakeholder groups across the globe (ABET, 2024), and as empathy can motivate engineers to respond in prosocial ways that are considerate of others (Hoffman, 2000), there is a need to understand how engineers empathize with others who are both near and far away. This need motivated the development of a framework depicting how empathy can manifest for stakeholders across six distinct frames of reference: (1) self, (2) team, (3) operators, (4) passengers, (5) bystanders, and (6) future generations. Each frame of reference incorporates a greater number of stakeholders and thus shifts from micro-ethical to macro-ethical in nature. Simultaneously, the scope of empathy shifts from self-oriented (self) to interpersonal (team) to systemic (operators, passengers, bystanders) to imaginative (future generations). Due to these shifts, cognitive empathy becomes increasingly primary in empathy’s activation or arousal.

**Fig. 1:**
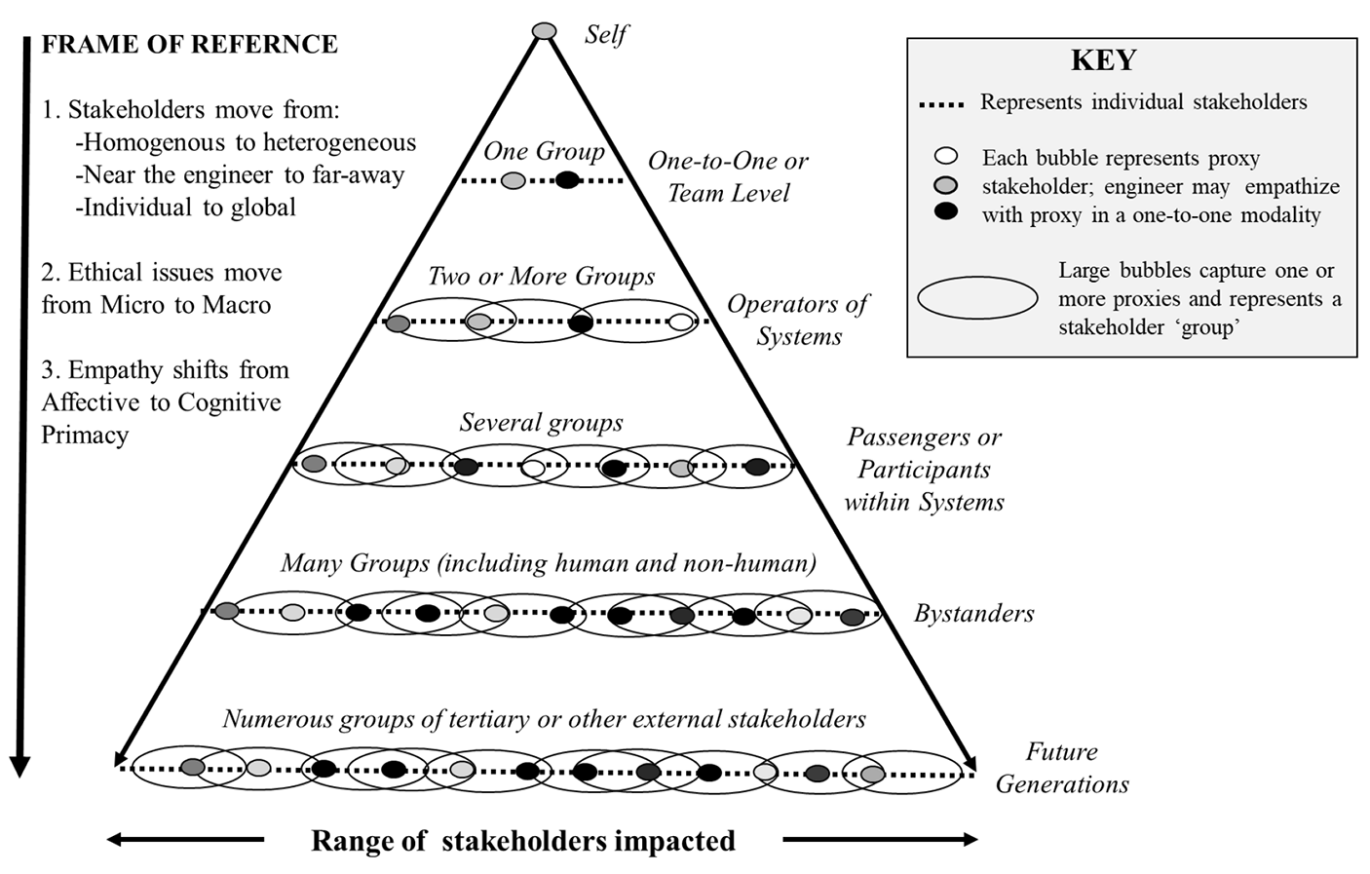
Depicting how the role of empathy shifts based on six frames of reference

Empathy starts with the self and the self persists through all frames of reference depicted in Fig. 1. The self is part of a team, and one’s self may also act as a system operator, passenger, bystander, or member of future generations (see Yong, 2016 for a take on the relationship between self-control and empathy for one’s future self). Focusing on cognitive empathy alone, imagine-self perspective-taking (a type of cognitive empathy) can inform imagine-other perspective-taking throughout other frames of reference. Imagine-other perspective-taking requires the individual to interpret another’s perspective through one’s own perspective and life history. As Hoffman (2000) describes, “although humans can empathize with the other, they are not the other” (p. 56). Thus, one must not lose sight of their self-perspective while engaging in imagine-other perspective-taking activities, as studies have shown when individuals blur the self-other boundary, their empathic accuracy declines (Decety & Jackson, 2004; Lawrence et al., 2006). Empathic accuracy itself will be situation-specific and will depend on a number of factors (Hoffman, 2000; Ickes, 2009), such as one’s affiliation with other groups (Allport, 1954).

The second level is the team level. At the team-level, micro-level considerations are a primary concern, wherein affective empathy (e.g., emotion congruence, empathic concern for peers) can promote feelings of inclusion, collective action, and positive interdependence, which (taken together) can lead to more positive outcomes. To this end, studies have sought to promote empathic formation within engineering teams (Akgün et al., 2015; Alzayed et al., 2020; Apfelbaum et al., 2021). Through direct interaction, affective empathy can manifest automatically. As an example, consider a team of four versus a group of forty; the former has greater potential to develop shared emotional congruence amongst all of its constituents. Moreover, when there is a lack of affective empathy, relatively small teams may operate in dysfunctional ways. For example, teams do not function well when members exhibit “negative interdependence” and lack “emotional intelligence,” (Luca & Tarricone, 2001), thus lacking understanding of and concern for peers’ feelings. In brief, affective empathy in particular may automatically manifest in team settings, and when it is absent it may be important to purposefully cultivate it to help teams realize their goals.

While the first two frames of reference depicted in Fig. 1 hearken to those individuals who are nearest to the engineer and whom they continuously directly interact with, the following four frames of reference name a more expansive set of stakeholder groups and thus identify stakeholder groups that are likely to be further away from the engineer. Whereas affective empathy is the primary modality leading to empathy’s manifestation at the team level, cognitive empathy becomes increasingly primary in the subsequent frames of reference. The final four frames of reference are borrowed and adapted from the victim classification scheme offered by Perrow (1999), who defined the four groups in his victim classification scheme as follows:

First-party victims are the operators; second-party victims are nonoperating personnel or system users such as passengers on a ship; third-party victims are innocent bystanders; fourth-party victims are fetuses and future generations. Generally, as we move from operators to future generations, the number of persons involved rises geometrically, risky activities are less well compensated, and the risks taken are increasingly unknown ones. (Perrow, 1999, p. 67).

Before depicting Perrow’s groups in more detail, it is worth articulating the salience of building on Perrow’s classification scheme here. Perrow’s objective was to delineate an exhaustive list of at-risk groups when designing high-risk technologies. As engineers craft such high-risk technologies, and as risks associated with such technologies are distributed throughout a global world, Perrow’s scheme is particularly useful, especially when compared to alternative stakeholder identification schemes that may focus more narrowly. For example, Wagner Mainardes et al. (2012) provided a summary of eight stakeholder classification schemes, none of which captured or emphasized global risks or impacts (p. 1864). Another example comes from Mitchell et al. (1997) who developed a model for managers within firms, wherein stakeholder groups were based on “(1) the stakeholder’s *power* to influence the firm, (2) the *legitimacy* of the stakeholder’s relationship with the firm, and (3) the *urgency* of the stakeholders’ claim on the firm” (p. 855); thus, like Wagner and Mainardes (2012), Mitchell’s (1997) scheme would lead to the deduction of stakeholders who are nearer to the firm. Finally, Scholes and Clutterbuck (1998) developed an “integrated approach” to communication between companies and stakeholders that accounted for all stakeholders who “have an influence on the key outcomes the company is looking for” and a vested interest in company pursuits (p. 228); again, this scheme emphasizes stakeholders who are near and directly impact a business or company. While these frameworks are valid for the purposes undergirding their design, they do not emphasize global impacts, which is a key part of the framework depicted in this paper.

According to Perrow (1999), “First-party victims are the operators of the system,” which include “those actually running the system” such as “plant operators” or “pilots,” as well as “first-level supervisors, maintenance personnel, low-level engineering personnel, and laborers and assisting personnel” (p. 67). While the team level in Fig. 1 depicts an engineer’s team or direct organizational group, the third frame of reference (i.e., operators) calls attention to the roles of other engineers and members of one’s organization who ensure system operations. Consider an engineer in pharmaceuticals whose primary role pertains to quality. The quality team itself may be the focus at the team level, but the same individuals and team can again reappear at the operator level. However, here this team will interface with other operational groups; in the context of pharmaceuticals, this may include design, development, manufacturing, or regulatory, among others. As operators, these stakeholders and stakeholder groups define and strive to uphold system-wide processes pertaining to their roles and responsibilities.

For Perrow, “Second-party victims are those associated with the system as suppliers or users but without influence over it” (p. 68). Such individuals may autonomously choose to be a part of an organization or system but may not wield decision-making power regarding the functioning of the system. Hence, Perrow framed this group as “passengers” or “participants” within the system. As participants, these individuals are (ideally) “aware of some risks” and act according to system processes. Should these individuals be unaware of risks pertaining to their roles, they may be relegated to “bystanders” (i.e., the following frame of reference). This line of argumentation aligns with Davis (1991), who suggested that some employees may lack “the information to evaluate the risk” associated with certain company-level decisions and thus may rather be depicted as bystanders (or, in Davis’s case, “part of the public,” p. 165).

The fifth frame of reference, bystanders, represents those who do not inform or engage in the operation of a system and do not actively assent to system operations. Such bystanders tend to be “innocent” in the sense that they do not wield decision-making power and, thus, would generally not be held responsible should accidents occur. Nonetheless, bystanders may be harmed when accidents occur. Perrow (1999) provided an example of residents living downstream from a dam whose property and livelihoods are at risk should the system fail. Like Perrow, Hoffman (2000) uses the term innocent bystanders, but in his work, Hoffman describes such bystanders as those who may “witness someone in pain, danger, or any other form of distress” (p. 29). Here, Hoffman is drawing attention to bystanders who are innocent in the sense that they are not responsible for the pain, danger, or distress they experience or observe in another. This is not to suggest that they are in no position to respond empathically.

Both Perrow and Hoffman describe a relationship between bystander status and blame. Perrow draws attention to how victims are often blamed for ethical mishaps by discussing nuclear power plants, which are often located near densely populated areas. While one might argue that those who live near a nuclear power plant knowingly choose to do so, many bystanders cannot choose to live elsewhere, or they may lack the information needed to adequately assess risks in this situation. Hoffman separately describes how “when people [i.e., bystanders] feel there is nothing they can do, they will often blame the victim as a way of distancing themselves and avoiding guilt” (p. 106). Hoffman suggests that promoting empathy among bystanders (specifically, empathy for victims) is essential to promoting helping behavior. 

In addition to prompting empathy for bystanders, both authors also call attention to motivating bystanders to empathize and then take action, especially for realizing ethical decisions that are responsive to victims. Perrow draws attention to the need for such empathic action when there is unfair distribution of risks associated with complex technology systems, particularly when these risks are inequitably received by those who do not uphold or are aware of the risks of such systems. Separately, Hoffman draws attention to the power that bystanders might wield to counteract unethical occurrences, and the role of empathy (particularly empathic distress) in promoting such responsiveness. In short, engineers should recognize that empathizing for bystanders is important, and when engineers are themselves “bystanders,” they may be poised (or perhaps even ethically obligated) to take action.

The final frame of reference calls for engineers to empathize with future generations. Perrow, continuing the nuclear power plant example, suggested that victims may include those not yet born who may be impacted by radiation. For such stakeholders, cognitive empathy is essential, since direct interaction with such stakeholders cannot occur (Hoffman, 2000). Perrow (1999) recognized the challenge and import of considering “inter-generational risks,” especially associated with high-risk technologies such as genetic modification and nuclear power. Given the imaginative nature of empathizing with future generations, here sustainable development frameworks can offer directed guidance for considering future generations (e.g., United Nations, 2015). Importantly, while future generations may be interpreted as those who are not yet born, a focus on generations may also include empathy for individuals currently inhabiting the earth, including oneself (Yong, 2016). Hence, even at this final frame of reference, the self persists.

In summary, as the frames of reference move from an individual to a global level, the proximity of the engineer to many impacted stakeholders decreases. As a result, engineers will not directly interact with all individual stakeholders, nor would empathizing with all individuals be feasible. Due to this infeasibility, empathy in engineering may shift from empathy-for-individuals to empathy-for-groups, thus making the focus of empathy more parsimonious and manageable. Yet, such a transition runs counter to how empathy (at least as traditionally conceived) manifests as a phenomenon. Thus, there is a need to provide accessible ways for engineers to empathize in a global world in purposeful and accurate ways. The frames of reference in this section draw attention to *who* engineers ought to empathize with, whereas the next section draws attention to *how* engineers may empathize with this broad range of stakeholders in effective way.

## Q5: How can Engineers Empathize with Stakeholders from the Self to Across the Globe?

The previous section offered six frames of reference for delineating stakeholders and stakeholder groups whom engineers ought to consider while making ethical decisions. This section provides guidance for the implementation of the framework, including defining stakeholder groups, identifying proxy stakeholders, and designing proxies (at least when needed).

As engineers must make ethical decisions in a global world, all six frames of references are relevant. Intentionally, Fig. 1 does not define the stakeholder groups within each frame. Thus, an engineer (or an engineering team or company) must answer the question, “Which stakeholder groups must I consider?” Potential groups associated with each frame of reference were briefly identified in the prior section (e.g., at the Operators frame, “plant operators,” “first-level supervisors, maintenance personnel, low-level engineering personnel, and laborers and assisting personnel,” Perrow, 1999, p. 67). However, depending on context, other stakeholder groups or more specificity may be required. For example, consider a company comprised of supervisors with important demographic variation: in such an instance, sub-groups may be pertinent for each of the groups listed here, such as supervisors with limited mobility and supervisors who are hearing impaired. This brief example serves to highlight that the pertinent groups and sub-groups engineers consider can vary by situation.

After defining stakeholder groups, the next step involves defining group proxies. Thus, an engineer must answer the question, “Which proxies effectively capture the needs or perspectives of a group?” Importantly, in Islind et al. (2023), “proxy users” refer to real individuals who can speak on behalf of users or a user group; here, proxy users are similar in the sense that proxies serve to represent a group, but distinct in the sense that proxies can be real or imagined. By defining or curating “proxy” stakeholders who represent stakeholder groups and purposefully empathizing with these proxy stakeholders, engineers can capitalize on empathy’s epistemic functions (Oxley, 2011). Moreover, by empathizing with an individual (i.e., a proxy), empathy manifests as one-to-one (rather than one-to-group), thus enabling engineers to capitalize on otherwise inaccessible empathy concepts (e.g., taking the perspective of a group is potentially impossible, and is certainly distinct from taking the perspective of an individual). The proxy stakeholders one chooses to represent a group may include a literal stakeholder. For example, if one wanted to design a nuclear weapon for the US military, their user proxy may be the current US president who, in the US, becomes the commander and chief during times of war. Often, however, a designer will need to craft a proxy stakeholder who effectively represents a stakeholder group. Design thinking strategies offer help here, particularly the user of personas.

Designers use personas to effectively (and empathically) design for user groups (Kouprie & Sleeswijk Visser, 2009). As stated by Adlin and Pruitt (2006), “Personas are detailed descriptions of imaginary people constructed out of well-understood, highly specified data about real people” (p. 3). Thus, persona development requires human-centered data collection (e.g., close observation, interviewing, immersion, surveys). While one can develop multiple personas to represent a group, there is – in theory – a primary persona who represents a group and who can become the target of empathy. As Cooper (2004) describes, “The primary persona is the individual who is the main focus of the design” and “who *must* be satisfied” (p. 137). Cooper adds, “If we find more than three primary personas, it means that our problem set is too large, and that we are trying to accomplish too much at one time” (p. 137). Reframed in terms of Fig. 1, if we find we have multiple primary personas for a single group, this may mean that there are multiple distinct stakeholder groups worth considering while making ethical decisions. 

The process of developing personas may reciprocally inform the stakeholder groups one must consider. Perrow (1999) provided an example of residents living near nuclear power plants that can help illustrate this point. A single user proxy may suffice for this group (i.e., residents), but it is possible there are important sub-groups (and, accordingly, multiple proxies) the engineer must consider. For example, there may be low-income residents who do not understand the risks associated with their choice of residence or who lack the resources needed to relocate. Separately, there may be high-income well-educated residents who actively resist and strive to halt the design, development, implementation, or operation of a nuclear power plant, such as in the case of Marble Hill (Chew, 2017). In this case, there may be activist-oriented residents and un-informed residents that may have distinct needs considering separately while proceeding in an engineering design process. 

To provide a concrete example of how personas can facilitate empathy, Cooper (2004) generated a set of personas representing potential customers of a User Interface for airlines, then identified a primary persona whom the design team should prioritize. This persona was named Clevis. Clevis was a 65-year-old “World Odyssey Class” airline passenger who “had no experience with computers and no patience for the typical attitude of delayed gratification that most programs have” (p. 144). Further, Clevis was an “aging” “Texan” who had “arthritis” and did not “own a computer or know how to use one.” As Clevis was the design team’s primary persona, by designing for Clevis, the team would theoretically satiate the needs of all “customers” (a stakeholder group). Proxy stakeholders like Clevis offer one way to bring empathy from a group-level to the one-to-one level, where it is more likely to initiate the “salience effect” described by Oxley (2011) or “empathic distress” as described by Hoffman (2000). When actual stakeholders cannot serve as representatives of a group, personas may become one’s focus. Indeed, even at this point, if you sought to empathize with Clevis, you are likely be able to do so to a greater extent than you would be able to for customers of “User Interfaces” on airlines, even though you know relatively little about Clevis.

## What are the Limitations of this Model and Areas of Future Work?

First, a critical question arises when using the framework offered in this paper: “Which groups must one consider?” This question is required for professional engineering practice. Consider the term “public,” which is central to many engineering codes of ethics (Davis, 1991); engineers use their discretion to define the “public” in appropriate (or, potentially, inappropriate) ways based on distinct situations (Lambrinidou et al., 2014; Smith, 2021). While Perrow’s work offers a starting point for defining stakeholder groups, instructors should center equity when deciding which individuals and stakeholder groups ought to be prioritized in engineering ethics, particularly given historic “equity silences” in engineering ethics (Rottmann & Reeve, 2020).

Second, a separate paper could be devoted to question five alone. When an actual individual is not a clear representative of a user group, personas offer a heuristic to discern and develop user proxies, but personas will serve as imperfect representations of stakeholder groups. The development of personas prompts one to develop an interpersonal empathy target, but empathic accuracy for the group will depend on the accuracy of the persona, as well as how representative the persona is of the group. Moreover, personas are *not* people and evaluating the accuracy of empathic judgements through direct user interaction will always be ideal, especially given that personas can de-humanize real stakeholders (Kwok-leung Ho et al., 2011).

Third, this writing was largely human-centric, especially considering its emphasis on understanding others’ cognitions and drawing inferences therefrom. The incorporation of innocent bystanders and future generations affords the opportunity to consider non-human stakeholders, but how empathy manifests may be dissimilar when the stakeholder or stakeholder group does not have a theory of mind (Doherty, 2012) or is a non-sentient being. In such instances, it is possible that a separate, cognitive-abled stakeholder may serve as the source for generalizing to non-cognitive stakeholders, but even this idea connotates a human-centric bias.

Finally, this paper did not describe factors undergirding engineers’ ethical or empathic formation. Prior empirical work has identified challenges in implementing empathy in engineering curriculums due to various factors, such as “distance, difference, and power” between the engineers and the others whom engineers strive to empathize with (Walther et al., 2020). Future work might utilize the model in curriculum to understand which stakeholder groups engineering students define and how dimensions such as these inform students’ identification or omission of stakeholder groups, selection of stakeholder groups, and identification or development of proxies.

## Conclusion

Empathy is a complex phenomenon and has distinct operationalizations inside and outside of engineering. Engineers must address macro-ethical issues in their work, including “global” issues (ABET, 2024). In this paper, affective empathy was described as having a primary role in microethics and an ethic of care whereas cognitive empathy was described as having a primary role in macroethics and justice. Building on these ideas, this paper offered a framework in the form of a pyramid structure to guide how one may empathize across six frames of reference, wherein the frames shifted from micro-ethical (i.e., self and team) to macro-ethical (i.e., primary, secondary, and global) foci. The framework depicted how group-level perspective-taking could occur by leveraging stakeholder proxies to facilitate macro-ethical reasoning for stakeholders beyond one’s immediate environments. This concerted emphasis on empathy and macroethics is novel but critical for helping engineers empathize with stakeholders across the globe. While proxy stakeholders might take the form of literal stakeholders, engineers might also employ design techniques to generate personas who, in turn, can become the “proxy stakeholders” for ethical decision-making. There are notable limitations associated with the framework and scholars should account for these limitations while using the framework in their research or teaching practices. By applying the framework, instructors can promote empathy in engineering ethics education to help students effectively integrate a comprehensive range of stakeholder needs and perspectives into their ethical decision-making processes.
